# Conceptualizing the Organization of Surgical Services Comment on "Decentralization and Regionalization of Surgical Care: A Review of Evidence for the Optimal Distribution of Surgical Services in Low- and Middle-Income Countries"

**DOI:** 10.34172/ijhpm.2020.60

**Published:** 2020-04-29

**Authors:** Sara A. Kreindler

**Affiliations:** Department of Community Health Sciences, Rady Faculty of Health Sciences, University of Manitoba, Winnipeg, MB, Canada.

**Keywords:** Surgery, Service Delivery, Regionalization, Decentralization, Low- And Middle-Income Countries (LMICs)

## Abstract

According to Iverson and colleagues’ thoughtful analysis, decisions to decentralize or regionalize surgical services must take into account contextual realities that may impede the safe execution of certain delivery models in lowand middle-income countries (LMICs), and should be governed by procedure-related considerations (specifically, volume, patient acuity, and procedure complexity). This commentary suggests that, by shifting attention to the mechanisms whereby (de)centralization may exert beneficial impacts, it is possible to generate guidance applicable to countries across the socioeconomic spectrum. Four key mechanisms can be identified: decentralization (1) minimizes the need for patients to travel for care and, (2) obviates certain system-induced delays once patients present; centralization (3) facilitates the maintenance of a workforce with sufficient expertise to offer services safely, and (4) conserves resources by limiting the number of sites. The commentary elucidates how context- and procedurerelated factors determine the importance of each mechanism, allowing planners to prioritize among them. Although some context factors have special relevance to LMICs, most can also appear in high-income countries (HICs), and the procedure-related factors are universal. Thus, evidence from countries at all income levels might be fruitfully combined into an integrated body of context-sensitive guidance.

Iverson and colleagues’ thorough review yields valuable guidance to countries seeking to optimize the organization of surgical services while working within resource constraints.^1^ The authors make a convincing argument that decisions to centralize or decentralize the delivery of a certain type of surgery should be informed by three considerations: the volume of patients, the acuity of the condition requiring surgery, and the complexity of the procedure. Another way to conceptualize the findings would be to understand centralization and decentralization as having distinct mechanisms of benefit, which are differentially important for different types of surgery and in different contexts. An advantage of this more abstract approach is that it allows the development of guidelines applicable to countries across the socioeconomic spectrum.

This commentary seeks to make explicit the key mechanisms whereby each model of service delivery can improve patient and system outcomes. I would suggest that it is possible to account for the review findings by positing just four mechanisms, two per model. Specifically, decentralization (1) minimizes the need for patients to travel for care and (2) obviates certain system-induced delays once patients present, while centralization (3) facilitates the maintenance of a workforce with sufficient expertise to offer services safely and (4) conserves resources by limiting the number of sites. As each model is associated with unique mechanisms of benefit, health systems face inevitable trade-offs. Fortunately, however, not all mechanisms are equally important for every procedure or in every context. By analyzing procedure and context factors in terms of their influence on the importance of each mechanism, it is possible to generate an integrated framework to govern decisions about service organization. Below I will attempt such an analysis, drawing on Iverson and colleagues’ findings from low- and middle-income countries (LMICs) alongside some evidence from high-income countries (HICs).

## Models of Service Organization and Their Mechanisms

Mechanism 1: Decentralization minimizes the need for patients to travel for care, allowing them to access services more quickly, conveniently, and inexpensively. Mechanism 1 seems key to the authors’ conclusion that decentralization is optimal for the treatment of high-acuity conditions (as travel time can delay care, putting patients at risk) and for high-volume procedures (as it is inefficient that large numbers of patients be required to travel). It also seems highly applicable to preventative services (which patients may choose not to access unless it is convenient to do so) and to services that demand comprehensiveness and continuity (eg, primary care, community-based care for chronic conditions), both of which the authors mention as potential domains for decentralization.^1^ Mechanism 1 would seem most relevant in contexts where the distances involved are large (ie, less so when centralization is a matter of reducing the number of facilities within the same city),^2,3^ transportation is difficult to access, and/or patients can ill afford the financial burden of travel.^1^ These contextual factors have obvious relevance to LMICs, but are also relevant to large HICs with remote communities.

Mechanism 2: Decentralization obviates certain system-induced delays that can occur after patients present, such as delays in transporting patients from outlying facilities to the appropriate referral centre. Like Mechanism 1, Mechanism 2 is most relevant to high-acuity and high-volume procedures, for the reasons of risk and inefficiency described above. It is most likely to apply in contexts where referral, transportation, communication and/or information systems are weak – hence, in LMICs.^1^ However, it is important to note that HICs are not immune to the problem of system-induced delay: In one Canadian study of the consolidation of acute-care surgery, patients who happened to present to a non-referral hospital waited significantly longer for their procedures.^4^ As the need for acute-care surgery, unlike some types of surgical need, may not be readily apparent, it may be difficult to ensure that patients present to the most appropriate site under a centralized system. Thus, one might add “easily diagnosed” to the list of characteristics of a surgical disease that make Mechanism 2 particularly relevant.

Mechanism 3: Centralization facilitates the maintenance of a workforce with sufficient expertise to offer services safely. This is not only because centralized models require fewer providers (making them easier to staff) but because they afford surgeons the opportunity to perform an adequate volume of each type of surgery to maintain their expertise.^5^ Mechanism 3 is key to the authors’ conclusion that centralization is most appropriate for procedures of low volume (least opportunity for regular practice) and high complexity (greatest need for expertise).^1^ Given the difficulty of establishing precise thresholds for volume–outcome relationships^5^ planners have some leeway to define “adequate volume,” and those in resource-limited contexts may choose a more liberal definition in order to balance the risk of inadequate volume against other risks. Planners in such settings are also more likely to explore the potential of task transfer to non-surgeons, which brings its own set of considerations about the development and maintenance of expertise.^1^ Nonetheless, Mechanism 3 in and of itself is important in LMICs as well as HICs.

Mechanism 4: Centralization conserves resources by limiting the number of sites that a system must maintain. (It might be noted that centralization and decentralization can have different resource implications depending on their specific features, such as the extent to which decentralization includes task transfer; all things being equal, however, centralized models should be less resource-intensive). Systems may be forced to centralize services in response to a shortage of surgeons (which frequently occurs in LMICs, as the authors note,^1^ but can also occur in HICs^4^), or may actively pursue economies of scale, including those associated with the creation of highly efficient centres for specialized, low-variability procedures (more likely to be undertaken in HICs).^6^ Resource constraints are, of course, more severe in LMICs; however, they may be a major motivator for centralization in HICs as well.^2^

It may also be useful to consider these mechanisms in light of the population–capacity–process model of service design.^7^ This model holds that, in order to design services that are well-aligned with population needs, planners must clearly define all populations in need of care and link each to appropriate capacity through an efficient process. We can observe that the mechanisms of decentralization are concerned with optimizing process (linking patients to care in as streamlined a way as possible), while those of centralization are concerned with optimizing capacity (ensuring the right type and quantity of resources, including providers, to meet patient needs). Although Mechanism 2 is also relevant to questions of population definition, as in the acute-care surgery example,^4^ most surgical populations are relatively easy to define. Thus, decisions to decentralize or centralize services typically involve a trade-off between optimizing process and optimizing capacity.

If decentralization is adopted to optimize process, alternative strategies may be required to ensure suitable capacity; as the authors note, workforce training is typically required, a single period of which may not suffice.^1^ Conversely, colleagues’ centralization is adopted to optimize capacity, alternative strategies may be required to compensate for process challenges. Mobile surgical camps may represent a means of ensuring local access to high-complexity (though not high-acuity) procedures; more broadly, centralization may necessitate major investments in referral, transportation and communication infrastructure.^1^ Early in the article, the authors raise the question of whether such investments would yield greater returns than the decentralization of services; through no fault of their own, they are unable to answer this question with the available data. However, it seems clear that all models of organizing services have some risks, whose mitigation might require a significant outlay of resources.

## Discussion

As the authors suggest, the “ideal distribution of services” is both procedure-specific and context-specific. Procedure factors determine the relative advantage of more rapid arrival *vs*. more expert provider – the former being most important for high-acuity surgery, the latter for low-volume and high-complexity surgery. Context affects the system’s ability to realize any of the mechanisms safely, through either service reorganization or alternative strategies. Thus, although the four identified mechanisms are relevant to both HICs and LMICs, context should shape decisions about which mechanism(s) to prioritize and how to operationalize them.

The premise underlying the authors’ undertaking to derive LMIC-specific recommendations is that evidence drawn from HIC-based studies may yield conclusions that are unsuitable for LMICs.^1^ At several junctures, the authors draw particular attention to the risks of centralization for LMICs that may not have the resources to execute it safely. Although none of the available studies of regionalization in LMICs uncovered adverse outcomes, such a concern remains legitimate: LMICs may, as a result of population characteristics and/or infrastructural limitations, be particularly vulnerable to the risks of regionalization. Nonetheless, rather than maintain two different sets of recommendations, it might be ideal to develop an integrated body of context-sensitive guidance, informed by findings from both LMICs and HICs. After all, all of the procedure-related and many of the context-related considerations identified through this review can also be relevant in HICs. Furthermore, the evidence regarding regionalization of surgical services in HICs is not monolithic; although centralization may be widely viewed as best practice, its impacts appear to vary by procedure and setting.^3,4,8-10^ The authors’ nuanced examination of evidence from LMICs reminds us how crucial it is that recommendations take adequate account of context. Perhaps guidance directed at health systems in general should look more like that offered by the authors^1^ – that is, perhaps what should be promoted is not *a model* of service delivery but rather a set of *principles and considerations for choosing*a model. Such an approach could potentially enrich decision-making in HICs and LMICs alike.

## Acknowledgements

I am grateful to Reena Kreindler for her editorial advice.

## Ethical issues

Not applicable.

## Competing interests

Author declares that she has no competing interests.

## Author’s contribution

SAK is the single author of the paper.
